# Is Intestinal Bacterial Diversity Enhanced by Trans-Species Spread in the Mixed-Species Flock of Hooded Crane (*Grus monacha*) and Bean Goose (*Anser fabalis*) Wintering in the Lower and Middle Yangtze River Floodplain?

**DOI:** 10.3390/ani11010233

**Published:** 2021-01-19

**Authors:** Zhuqing Yang, Lizhi Zhou

**Affiliations:** 1 School of Resources and Environmental Engineering, Anhui University, Hefei 230601, China; x18201023@stu.ahu.edu.cn; 2 Anhui Province Key Laboratory of Wetland Ecological Protection and Restoration, Anhui University, Hefei 230601, China

**Keywords:** intestinal bacteria, trans-species, pathogen, interaction, migratory waterbirds

## Abstract

**Simple Summary:**

Intestinal microbes play an indispensable role in host physiology and their alteration can produce serious effects on vertebrates. In this study, we analyzed the characteristics of intestinal bacterial community of hooded crane and bean goose whose niches overlap at Shengjin Lake, China, and investigated how host internal factors and inter-species interactions affected the diversity and spread of intestinal bacteria of the two species over three wintering periods. We have found that direct or indirect contact with each other increased the diversity of host intestinal bacteria and caused bacteria to spread among species in the mixed-species flock. In addition, a total of 63 pathogens were identified, of which 38 (60.3%) were found in the gut of both species. These findings could help our understanding of the factors that influence gut bacteria in wild waterbirds, which are also major contributors to the spread of pathogens worldwide.

**Abstract:**

Diversity of gut microbes is influenced by many aspects, including the host internal factors and even direct or indirect contact with other birds, which is particularly important for mixed-species wintering waterbird flocks. In this study, Illumina high-throughput sequencing was used to analyze the intestinal bacteria of the hooded crane and bean goose whose niches overlap at Shengjin Lake. We tested whether contact time enhances the trans-species spread of gut bacteria. Results indicate alpha-diversity and microbial composition displayed significant separation between the two hosts in every wintering period, although the number of bacteria types shared increased with increasing contact time. For the same species, with the lengthening of contact time, alpha-diversity and the number of operational taxonomic units (OTUs) in the host intestine augmented, and the common OTUs and structural similarity of microflora in the middle and late periods were more than in the early and middle periods. In addition, we found a very high proportion of shared pathogens. Our results indicate that, although intestinal microflora of different species were separated, direct or indirect contact in the mixed-species flock caused the spread of gut bacteria trans-species, indicating that more attention should be paid to intestinal pathogens in wild birds.

## 1. Introduction

Intestinal microbes can co-evolve with the host, playing an indispensable role in host physiology. Changes in gut microbes can produce serious effects on vertebrates, for example, increasing susceptibility of the host to diseases [1]. However, many internal host factors and external environmental factors may alter the structure of the intestinal microbiota. Many studies have confirmed that the host is a key factor affecting intestinal microorganisms [2,3,4,5]. For example, a study revealed significant differences between the cloacal microorganisms of parasitic nestlings and host nestlings by experimentally creating mixed broods of magpies (*Pica pica*) and great spotted cuckoos (*Clamator glandarius*) [6]. Despite differences in the gut microbes of different sexes and ages, greater similarity occurs between gut microbes of the same species than in different species, with inter-group differences always being significantly greater than intra-group differences [7,8]. The environment and genotype of the host determine whether a microbe can survive and reproduce in the gut [9]. After being ingested, most of the external bacteria are eliminated in the host, with only a few remaining [10].

However, not only are enteric microorganisms affected by the host itself, but the environment can also result in high plasticity, and complex environments will increase the diversity of intestinal microorganisms [11]. Studies have shown that bacteria from different sources colonize and persist in the gut of the same host, and some perform specific biological functions [12]. Therefore, it is possible to spread intestinal microbes from one host to another, rather than spreading vertically within the host [13,14]. Gut microbes from different species are spread either directly by physical contact or indirectly through vectors such as soil [15]. This is especially relevant for mixed-species flocks with overlapping niches, which can enhance the connection between microbial communities and bacterial trans-species horizontal transmission by sharing the same habitats and food resources [16,17], generating similarity in intestinal bacterial diversity. The trans-species spread will allow the host to acquire more diverse and complex bacteria from other species, and to some extent, increase the host′s resistance to pathogenic bacteria [18]. However, it will also facilitate the spread of pathogenic bacteria among sympatric hosts, increasing the difficulty in the prevention and control of infectious diseases.

Migrating waterbirds live in groups and often flock in mixed species, resulting in overlapping habitats and feeding grounds [19,20]. Migratory birds with a complex travel pattern increase the chance of carrying different pathogens from various regions, as well as the diversity of gut microbes [21]. In addition, wild birds are natural carriers of many pathogens [22], and use the same habitat for foraging, breeding, and defecating. Through contact with other birds, as well as food, soil, air, water, and other transmission media, other birds in the sympatric domain get infected with the microorganisms [19,20,23]. The effect these contacts on the gut microbial structure remains relatively unknown and is to be explored, as it poses great significance for the protection of birds and the prevention and control of infectious diseases.

The hooded crane (*Grus monacha*) is a vulnerable (VU) species according to the International Union for Conservation of Nature Red List of Threatened Species, first class of key protected animal species of China, and a flagship species in wetland ecosystems. Hooded cranes breed in the far east of Russia and the northeast of China every year, which augments the likelihood that they carry bacteria, including pathogens, from different regions. In autumn, hooded cranes migrate to the middle and lower Yangtze River floodplain for wintering, and Shengjin Lake, a typical river-connected sallow lake in the lower and middle Yangtze River floodplain, is their important wintering ground in the mainland [24]. As a large wader, hooded cranes winter in clusters [25], and their wintering habitats are relatively fixed. As hooded cranes are relatively rare, their gut microbes may be more susceptible to other birds with larger populations. Bean goose (*Anser fabalis*) is another large winter migratory bird species; its winter migration is in clusters, which are widely distributed and abundant. It is estimated that as many as 20,000 bean geese winter in China every year, with 60% of the population using the migration route at Shengjin Lake [26]. Hooded cranes and bean geese wintering at Shengjin Lake share the same niche, and the degree of overlap increases with the advance of the wintering period and the lengthening of contact time [27]. With the continuous lengthening of the contact time, the contact degree of hooded crane and bean goose is enhancing.

The fecal microbiota structure of migratory birds has high temporal stability [28]. However, if gut microbes are spread from host to host, the intestinal bacteria of mixed-species flock will change, showing the same or similar change patterns. In this study, we characterized gut microbiota and the potential pathogenic bacteria in hooded cranes and bean geese at Shengjin Lake through high-throughput sequencing and bioinformatics analysis. We propose the following hypotheses: (a) bird species differences can lead to significant discrepancies in intestinal bacterial communities; (b) contact leads to horizontal transmission of intestinal microflora between different species; (c) with the lengthening of contact time, the gut bacteria of hooded cranes and bean geese will change and show the same variation pattern during the wintering period.

## 2. Materials and Methods

### 2.1. Ethics Statement

All samples in this study were collected by the non-damage sampling method (fecal sampling method). We waited until the birds had finished their feeding period to collect the fecal samples, and no birds was disturbed or harmed in this procedure. We obtained permission from the administrative department of Anhui Shengjin Lake National Nature Reserve, China to collect samples.

### 2.2. Site Selection and Fecal Sample Collection

Shengjin Lake (116°55′–117°15′ E, 30°15′–30°30′ N) located in the middle and lower Yangtze River floodplain, is a Ramsar site [29]. The wintering period of the hooded crane at Shengjin Lake was divided into three stages: the early period (from the time of moving to December), middle period (January to February), and late period (from March to the time of moving out) [30,31]. Studies have shown that the niche of the hooded crane and the bean goose overlap, and the degree of overlap increases with the advance of the wintering period at Shengjin Lake [27]. We chose the upper side of Shengjin Lake, where hooded crane and bean goose forage in mixed-species flock, as the research site to collect samples.

We collected fecal samples of mixed-species, hooded cranes, and bean geese in the early period (time: 23 November 2018; habitat: grass land), middle period (time: 9–10 January 2019; habitat: hooded cranes were in rice field, bean geese were in grass land), and late period (time: 8 March 2019; habitat: grass land). Samples of both species were collected at one location, and there were about 100 hooded cranes and about 300 bean geese at each period to eliminate interference from other factors as much as possible (Supplementary Table S1 and Figure S1). First, we observed the species and number of birds with a telescope, and populations with more than 80 individuals were selected. Fresh feces were collected in clean zipper bags and using a pair of polyethylene gloves, which was replaced for each sample to prevent mutual contamination. In addition, samples were collected at an interval distance of 5 m to avoid repetition in sampling, touching the ground was avoided, and the middle portion of the sample was collected. After collection, feces were stored in an incubator with ice bags for short-distance storage, promptly brought back to the laboratory, and stored in an ultra-low temperature refrigerator at −80 °C. A total of 109 samples were used in this study. Among them, the number of samples from hooded cranes in the early period (HCE), middle period (HCM), late period (HCL) was 20, 20, and 17, respectively. The number of samples from bean geese in the early period (BGE), middle period (BGM), late period (BGL) was 15, 19, and 18, respectively.

### 2.3. Fecal DNA Extraction

Fecal DNA was extracted using the fecal DNA extraction kit (QIAamp Fast DNA Stool Mini Kit, MP Biomedicals, Santa Ana, CA, USA), quantified using a micro-spectrophotometer (NanoDrop ND-1000, Thermo Fisher Scientific, Waltham, MA, USA) and stored at -20 °C for post-order analysis.

### 2.4. Bird Species Determination

The mitochondrion COI barcode area was amplified to confirm whether the DNA samples belonged to the hooded crane or bean goose. We used primers F1-R1 (F1:TTCTCCAACCACAAAGACATTGGCAC; R1:ACGTGGGAGATAATTCCAAATCCTG) to identify hooded crane [32], and primer B1-G1 (B1:CTCATCTTCGGGGCATGAGC; G1:GAATAGGTGTTGGTACAGGATTGG) to identify bean goose. A reaction system of 50 μL was prepared as follows: 25 μL SuperMix [2 × EasyTaq^®^PCR SuperMix(+ dye), TRANSGEN, Beijing, China], 1 μL DNA template, 1 μL forward primer (10 μM), 1 μL reverse primer (10 μM), and 22 μL nuclease-free water. PCR parameters were as follows: 5 min at 95 °C; 30 s at 95 °C, 45 s at 55 °C, 90 s at 72 °C, and 35 cycles; 10 min at 72 °C for final extension. The final product was subjected to gel electrophoresis, and samples with clear strips in the ultraviolet analyzer were sent to Sangon Biotech in Shanghai for sequencing. The sequencing results were compared on the National Center for Biotechnology Information, Bethesda, MD, USA. The DNA of the hooded crane or bean goose (similarity > 97%) was preserved for high-throughput sequencing.

### 2.5. Illumina MiSeq Sequencing

Purified DNA (30 μL) was extracted from each sample as an amplification template and sent to the Illumina Mi-seq platform of Shanghai Meiji Biological Company. The V4-V5 hypervariable region of the bacterial 16S rRNA gene fragment was amplified with primers F515/R907 (Supplementary Table S2).

### 2.6. Processing of Sequenced Data

The raw sequencing data were pre-processed by QIIME (V.1.9) [33]. Low-quality sequences with a sequence length ≤ 250 bp, or an average quality score < 30, were eliminated. UCLUST was used to cluster high-quality sequences into operational taxonomic units (OTUs) with 97% similarity [34]. Chimeras and singleton OTUs were deleted. The most abundant sequence in each OTU was selected as the representative sequence and identified with the ribosomal database project Classifier [35]. Representation was aligned using PyNAST [33]. We selected 8000 sequence subsets (minimum sequence read depth; repeat 20 times) randomly, to equally rarefy samples, for comparing similarities and differences in bacterial community composition. Chloroplasts and Archaea were outside the scope of our analysis. The raw data have been submitted to the NCBI Sequence Read Archive (accession number SRP292607).

### 2.7. Identification of Potential Pathogenic Bacteria

All the identified bacterial species were searched through the Web of Science to identify pathogenic bacteria or potentially pathogenic bacteria. Bacteria that have been demonstrated to be pathogenic to humans, animals, or plants, were sorted out for special analysis and all have corresponding literature support.

### 2.8. Biostatistical Analyses

Linear discriminant analysis effect size (LEfSe) was used to analyze the differences in intestinal bacterial groups in the two host species, using the non-parametric Kruskal-Wallis rank sum test with the setting (an α value of 0.05 and an effect size threshold of 2.3) to identify biomarkers [36]. Differences in intestinal bacterial community composition among different hosts and at different periods were analyzed by non-metric multidimensional scaling (NMDS) and analysis of similarity (ANOSIM; permutations = 999), which use the vegan package (V.2.0-2) in RStudio (V.1.1.463). Indicators of intestinal bacteria in each group were analyzed using indicator analysis with the Labdsv package in RStudio. The contribution of each bacterial species to the total difference in the bacterial flora of the two species and three periods was analyzed by SIMPER analysis routine in RStudio [37]. The alpha-diversity and relative abundance of dominant bacteria (>1%) were analyzed by one-way analysis of variance (ANOVA) for data that conformed to a normal distribution (Kolmogorov-Smirnov test, *p* > 0.05), or by Mann–Whitney U test for data that conformed to a non-normal distribution (Kolmogorov-Smirnov test, *p* < 0.05) (Supplementary Table S3).

## 3. Results

### 3.1. Differences in Intestinal Bacterial Diversity between Hooded Crane and Bean Goose

We obtained a total of 3,061,103 high-quality sequences from the hyper-variable V4–V5 region of the 16S rRNA gene, with the number of sequences per sample ranging from 8026 to 58,336 (Supplementary Table S4). A total of 25,655 bacterial OTUs with 97% sequence similarity and 42 bacterial phyla were identified. The common OTUs of the two species were 8979 (35%), and the unique OTUs of the hooded cranes and the bean geese were 11,414 (44.5%) and 5262 (20.5%), respectively (Supplementary Figure S2). The alpha-diversity of intestinal microflora in the two species was evaluated by OTU richness, phylogenetic diversity, and the Chao1 index. One-way ANOVA showed that the alpha-diversity with three indicators of the hooded cranes gut bacteria was significantly higher than that of bean geese (*p* < 0.001) (Supplementary Figure S3).

**Figure 1:**
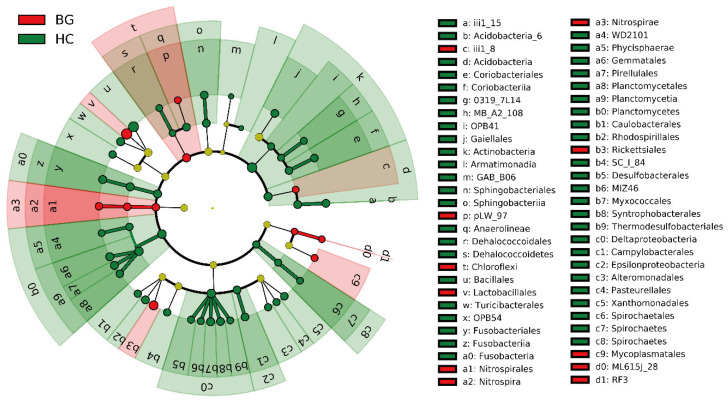
LEfSe analysis of gut bacterial biomarkers of the hooded crane and bean goose. The identified phylotype biomarkers were ranked by effect size and the alpha value was < 0.05. Cladogram representing the taxonomic hierarchical structure from phylum to order of the biomarkers identified in the hooded crane and bean goose; red, phylotypes overrepresented in the gut of the bean goose; green, phylotypes overrepresented in the gut of the hooded crane; yellow, phylotypes which relative abundance is not significantly different between the two host types. HC: hooded crane; BG: bean goose.

The dominant bacterial phyla of intestinal bacteria were Firmicutes, Proteobacteria, Actinobacteria, and Bacteroidetes in the two hosts. Actinobacteria contributed more to the gut microbiota of hooded cranes (8.28 ± 6.76%) than to bean geese (5.86 ± 6.15%), and the other dominant phyla showed no significant differences between the two species (Table 1). LEfSe analysis identified specific taxa of gut bacteria with significant differences in abundance between the two species. The results showed that the abundance of bacteria in the gut of hooded cranes was significantly increased in 5 phyla, 14 classes, and 28 orders, while that of the bean geese was significantly increased in 2 phyla, 3 classes, and 6 orders (Figure 1 and Supplementary Figure S4). Analysis of similarity proved that there was a significant difference in intestinal bacterial community structure between hooded cranes and bean geese (ANOSIM, *p* = 0.001; Table 2), suggesting that the hooded cranes have a richer intestinal bacterial community than the bean geese.

### 3.2. Intestinal Bacterial Diversity and Its Diversity Patterns in Hooded Cranes and Bean Geese

**Figure 2:**
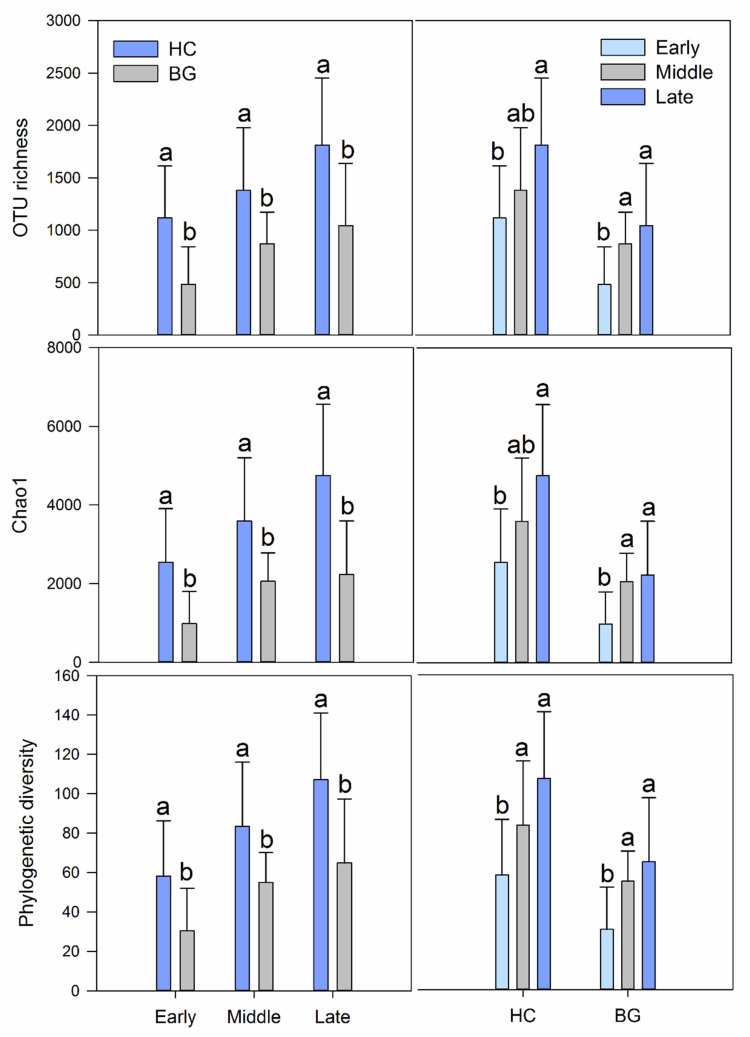
Intestinal bacterial alpha-diversity in different hosts (**left**) and different periods (**right**). Bars represent means; error bars denote standard deviations; Differences in intestinal bacterial alpha-diversity between different species were identified by one-way ANOVA (*p* < 0.01). Differences in intestinal bacterial alpha-diversity between different periods were identified by Tukey HSD. OTU: operational taxonomic unit.

Identification of the intestinal microflora was performed and compared between hooded crane and bean goose in each wintering period. In the early, middle, and late period, OTUs of mutual contained bacteria among the two hosts were 2313 (23.2%), 3422 (22.9%), and 4112 (24.1%), respectively. In the early, middle, and late period, the OTUs unique to the hooded cranes were 6033 (60.5%), 8318 (55.7%), and 7730 (45.3%), respectively. In the early, middle, and late period, the OTUs unique to bean geese were 1619 (16.2%), 3204 (21.4%), and 5224 (30.6%), respectively (Supplementary Figure S5). Therefore, the types of bacteria shared by the two hosts increased with the lengthening of contact time. In addition, the OTUs of hooded cranes were more abundant than those of bean geese in all periods. At any period, the alpha-diversity of gut bacteria of hooded cranes was significantly higher than that of bean geese (*p* < 0.01; Figure 2 and Supplementary Table S5).

In addition, we identified and compared the intestinal microflora diversity patterns during different periods of wintering in the same species. The same pattern was observed in the gut bacterial communities of hooded cranes and bean geese. In the early, middle, and late period, 8346, 11,740, and 11,842 bacterial OTUs, respectively, were found in the intestines of hooded cranes and 3932, 6626, and 9336 OTUs, respectively, were found in the intestines of bean geese. For hooded cranes, OTUs shared between the three periods were 14.5% (2967), HCE and HCM were 21.1% (4323), and HCM and HCL were 29.6% (6038), and for bean geese, they were 7.8% (1111), 11.2% (1591), and 23.8% (3394), respectively. With the increase in contact time, the number of bacterial OTUs in the host intestine was increased, and the common OTUs (number and proportion) in the middle and late periods were more than in the early and middle periods, which was observed in both hooded cranes and bean geese. In both hooded cranes and the bean geese, the alpha-diversity of intestinal bacteria was increased, but discrepancy in alpha-diversity was decreased throughout the wintering period (Figure 2).

**Figure 3:**
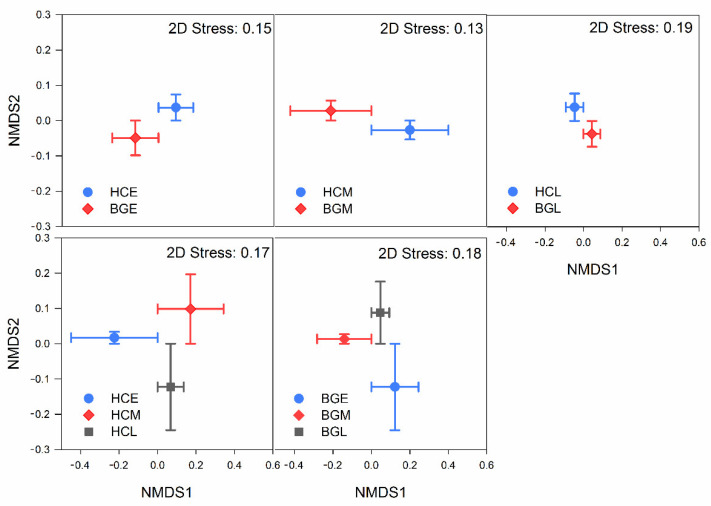
Community structure of gut bacteria in different species (**top panel**) and different periods (**low panel**). NMDS: non-metric multidimensional scaling; HC: hooded crane; BG: bean goose; E: early period; M: middle period; L: late period.

Differences in intestinal microflora composition among different hosts were assessed via NMDS and analysis of similarity, showing that the microbial community structure displayed significant separation between host species in every period (Figure 3 and Table 2). The contribution of taxa to the intestinal microflora structure discrepancy between the hooded cranes and bean geese in every period was identified by SIMPER analysis, revealing that *Lactobacillus*, Peptostreptococcaceae, *Solibacillus*, *Exiguobacterium*, and *Streptococcus* made a significant contribution to the structural difference of intestinal microflora in the early period. *Lactobacillus*, Enterococcaceae, Peptostreptococcaceae, *Bacillus*, and *Agrobacterium* contributed 46.22% to the composition of bacterial flora in the middle period. *Lactobacillus*, *Clostridium*, *Solibacillus*, *Bacillus*, and *Fecalibacterium prausnitzii* were the five most vital biological groups accounting for the difference in late period (Table 3). According to the analysis of indicators, in all bacterial groups with relative abundance greater than 0.5%, 8 bacterial taxa (6 pertaining to HCE; 2 belonging to BGE) were identified in the early period, 11 in the middle period (8 pertaining to HCM; 3 belonging to BGM), and 11 in the late period (8 pertaining to HCL; 3 belonging to BGL) (Supplementary Table S6) (BGE, samples from bean geese in the early period; BGM, samples from bean geese in the middle period; BGL, samples from bean geese in the late period; HCE, samples from hooded cranes in the early period; HCM, samples from hooded cranes in the middle period; HCL, samples from hooded cranes in the late period).

The gut microflora composition patterns during different periods in the same species were also explored. The early and middle periods showed greater separation than the middle and late periods (Figure 3 and Table 2). SIMPER analysis revealed that *Lactobacillus*, Peptostreptococcaceae, *Solibacillus*, Nitrosomonadaceae, and *Paenibacillus* made a key contribution to the structural differences of intestinal microflora in HCE and HCM. *Lactobacillus*, *Solibacillus*, *Bacillus*, *Agrobacterium*, and *Paenibacillus* contributed 33.90% in HCM and HCL. *Lactobacillus*, *Solibacillus*, Enterococcaceae, Peptostreptococcaceae, and *Streptococcus* contributed 39.75% in BGE and BGM. *Lactobacillus*, Enterococcaceae, *Clostridium*, *Streptococcus*, and Peptostreptococcaceae were the five most vital biological groups accounting for the differences of BGM and BGL (Table 3). Indicator analysis was carried out to identify bacteria associated with the wintering period; consequently, it identified 16 indicator taxa (10, 2, and 4 in HCE, HCM, and HCL, respectively) for hooded cranes, and 12 indicator taxa (6, 2, and 4 in BGE, BGM, and BGL, respectively) for bean geese (Supplementary Table S6).

### 3.3. Variation Characteristics of Early Period Bacteria in Hooded Crane and Bean Goose during the Wintering Period

**Figure 4:**
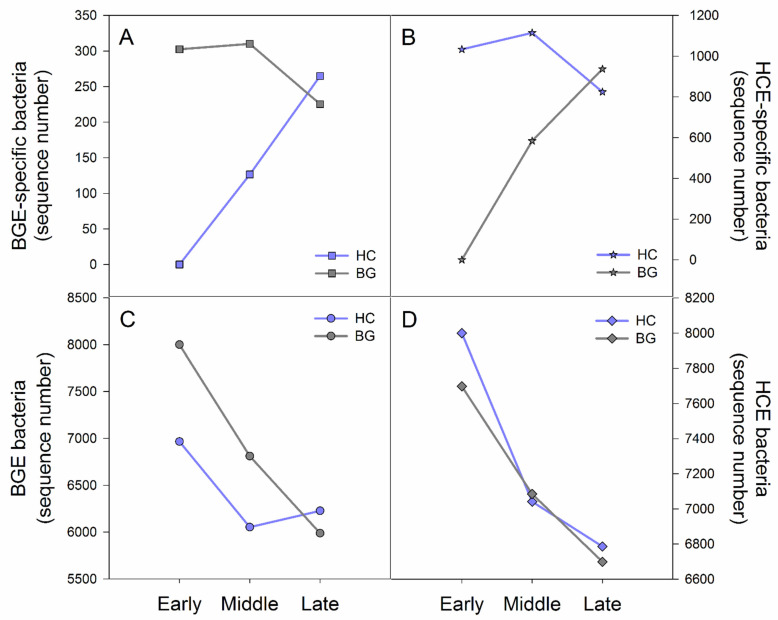
Variation of early period bacteria in hooded crane and bean goose during the wintering period. (**A**) BGE-specific bacterial sequence number in the two species and three periods; (**B**) HCE-specific bacterial sequence number in the two species and three periods; (**C**) BGE bacterial sequence number in the two species and three periods; (**D**) HCE bacterial sequence number in the two species and three periods. The horizontal axis represents the wintering period and the vertical axis represents the average number of sequences. HC: hooded crane; BG: bean goose; E: early period.

To better understand the transmission of intestinal bacteria between the two species, we analyzed their variation in HCE and BGE in the three periods. With the lengthening of contact time, BGE-specific bacteria (existing in BGE but not HCE) appeared in the gut of hooded cranes, and HCE-specific bacteria (existing in HCE but not BGE) appeared in the gut of bean geese. Additionally, the number of average sequences of two specific bacteria in both species became increasingly similar (Figure 4 and Supplementary Table S7), which suggested that the HCE-specific or BGE-specific bacteria were being transmitted from host to host through contact. Furthermore, the average sequence of BGE bacteria in the gut of the two hosts also showed increasing characteristics that were similar to the specific bacteria. However, HCE bacteria in both hosts were similar at every period (Figure 4 and Supplementary Table S7), suggesting that HCE bacteria may be the basic bacteria prevalent in the intestines of birds. Thus, gut bacteria in hooded cranes and bean geese were influenced by each other, but bean geese had a greater influence on hooded cranes.

### 3.4. Intestinal Potential Pathogens in Hooded Cranes and Bean Geese

**Figure 5:**
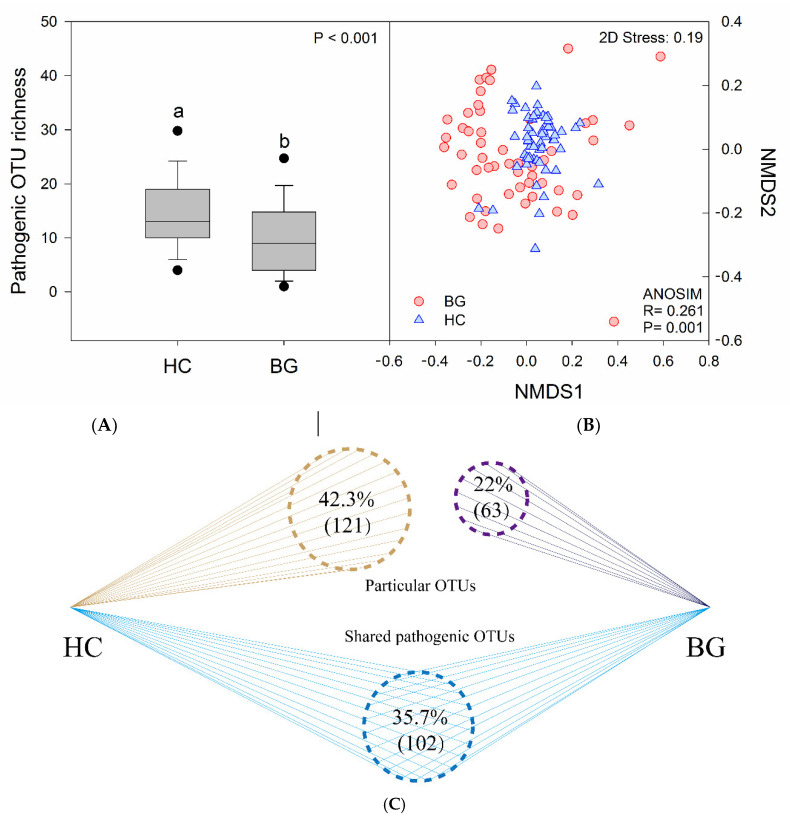
Intestinal pathogenic bacterial characteristics in the hooded crane and bean goose. Pathogenic operational taxonomic unit (OTU) richness (**A**), pathogenic bacterial community composition (**B**), pathogenic OTU overlapping (**C**). NMDS: non-metric multidimensional scaling; HC: hooded crane; BG: bean goose.

We detected that pathogens were present in all samples, and a total of 8331 potential pathogen sequences were found in the intestines of both species, ranging from 1 to 883 sequences per sample, and occupying 0.955% of total bacterial sequences read (Supplementary Table S4). Among them, the pathogen sequences in the guts of hooded cranes were 3615 (0.793%) and in those of bean geese were 4716 (1.134%). Of the 286 pathogenic OTUs identified, 35.7% (102) were common among the two hosts, 42.3% (121) were specific for hooded cranes, and 22% (63) were specific for bean geese (Figure 5). The potential pathogen OTU richness of hooded cranes was significantly higher than that of bean geese (one-way ANOVA, *p* < 0.001) (Figure 5).

A total of 63 pathogens were identified, of which 38 (60.3%) were found in the gut of both hosts (Supplementary Table S8). The pathogenic bacteria with the highest content was *Agrobacterium vitis*, accounting for 42.573% of the total pathogen sequence in hooded cranes, which can cause crown gall disease of wine grapes [38]. *Clostridium perfringens* was the most abundant animal pathogenic bacterium in hooded cranes, accounting for 17.538% of pathogenic bacteria identified, which can cause diseases such as tissue necrosis and bacteraemia in humans and birds. *Enterococcus cecorum* was the most prevalent pathogen in bean geese (63.253%), posing a pathogenic threat to humans, birds, poultry, and other organisms. NMDS and ANOSIM showed differences in the composition of potential pathogens in the intestines of hooded cranes and bean geese (R = 0.261, *p* = 0.001) (Figure 5).

## 4. Discussion

Whether host self-factors or external environmental factors exert greater influence on gut microbes is a controversial topic. In our study, the effects of host self-factors and external environmental factors on intestinal microflora were investigated simultaneously, and the trans-species spread of mixed-species flock intestinal bacteria was further analyzed. The niche of the hooded crane and the bean goose at Shengjin Lake overlap, and the degree of overlap is increased with the advance of the wintering period [27]. As the contact time between hooded cranes and bean geese increased, the types of bacteria shared by the two hosts increased, the number of bacterial OTUs in each species was higher, and the common OTUs (whether number or proportion) in the middle and late periods of wintering were more than those in the early and middle periods, either in hooded cranes or bean geese, which proved the influence of external environmental factors on the host intestinal bacteria OTUs without exception. Likewise, alpha-diversity also reflects the effects of the environment on gut bacteria based on the wintering period (Figure 2). Alpha-diversity represents the richness and evenness of species in a community and is the most intuitive way to compare communities. As the contact time increased, alpha-diversity increased and the discrepancy in alpha-diversity decreased throughout the whole wintering period, the microflora structure of the middle and late periods was more similar than that of the early and middle periods, for the same host (Figure 2 and Table 2). An increase in the overlapping niche increases the likelihood of transmission of bacteria between the two hosts. The increase in abundance of gut bacteria in hooded cranes and bean geese followed like an increasing logarithmic curve, and the two species showed the same variation pattern during the wintering period. The fecal microbiota structures of migratory birds have high temporal stability [28]. Furthermore, previous studies on the intestinal bacteria of the hooded crane showed that the phylogenetic diversity index and OTU richness had no significant difference among the three periods at Shengjin Lake [30]. However, in our study, the hooded cranes and the bean geese showed a similar variation pattern, which reveals that contact leads to the trans-species spread of gut microbes in mixed-species flocks. Migratory birds are sensitive to environmental changes, so shared environments promote similarity in microbial communities. Previous studies of hooded cranes have shown that the alpha-diversity of intestinal bacteria varies in different seasons and the composition of intestinal microbial communities varies in different stopping places [39]. Individuals from the same environment contain more similar microbial communities than individuals from different environments [8]. It has been reported that social behaviour and shared environment promote the horizontal transmission of microorganisms between different hosts [40], which is consistent with our findings.

However, based on the species-level analysis, significant differences were found in both the alpha-diversity (*p* < 0.01; Figure 2) and the composition of bacterial communities (*p* < 0.001; Table 2) in the gut microflora of hooded cranes and bean geese. Although direct or indirect contact with each other can lead to the trans-species spread of intestinal bacteria, the two different hosts remained significantly different in the three wintering periods, indicating that host factors have more influence on intestinal bacteria than environmental factors. It should be noted that although the middle period samples of hooded cranes were collected in the paddy fields, the same pattern was observed in the gut bacteria of hooded cranes and bean geese, which indicated that the influence of food factors was insignificant in our study. In addition, the specific dietary classification is meaningless compared with the broad dietary classification (for example, herbivorous, carnivorous, and omnivorous) [41], so it was not repeated here. Although many studies have shown little evidence of a correlation between geographic distance and microflora similarity [4,41,42], we collected samples from hooded cranes and bean geese that lived very close to each other to ensure the precision of the experiment. Significant differences in intestinal bacterial communities of sympatric hooded cranes and domestic geese with strong host preference for gut microbial flora have been observed in previous studies [43], but no comparative study on the influence of external factors is available. Colonization of gut bacteria in a bird begins at birth, as the initial bacteria spread vertically rather than horizontally. Pioneer gut colonizing species may have influenced later species. In addition, host internal factors can also influence which bacteria can survive or reproduce in the gut, while external environmental factors can determine which bacteria can enter the host gut.

In this study, hooded cranes had a higher diversity of gut bacteria and more representative bacterial taxa than bean geese, probably because the population size of hooded cranes is relatively small, and thus, more susceptible to other species. There are only a few hundred hooded cranes [44] and as many as tens of thousands of bean geese at Shengjin Lake, so the hooded cranes are more influenced by the bean geese (Figure 4). However, the greater diversity of gut bacteria in hooded cranes might have been influenced by other species apart from the bean geese.

Glycosides are the storage form of most plant polyphenols in food [45], while Firmicutes and Bacteroidetes play a key role in the decomposition of polysaccharides [46]; Bacteroidetes can also hydrolyze proteins [45], playing a key role in the breakdown of food, such as plant cell walls and starch granule. There was no significant difference in the dominant phylum between the two species, and Actinobacteria showed only a small difference (*p* = 0.049; Table 1), which suggests that the hooded crane and bean goose have a similar dietary structure at the micro level. Previous studies have come to similar conclusions at the macro level; rice grains and wheat seedlings are the main food components of bean goose during the wintering period [26], as well as the hooded crane [30,47].

We also detected a large number of pathogenic bacterial sequences in the intestines of hooded cranes and bean geese, accounting for 0.955% of the total bacterial sequences read. The 63 pathogens identified can cause a variety of diseases in humans, animals, and plants (Supplementary Table S8). It is noteworthy that *Paracoccus aminovorans* is able to promote the growth of *Vibrio cholerae* [48], found in both hooded cranes and bean geese. A previous study indicated that there is a negative correlation between bacterial community and pathogenic microbes [2]. Our study also suggests that gut microbiota can inhibit pathogen colonization, as hooded cranes had more pathogenic OTUs than bean geese with lesser sequences and lower proportion of pathogens. Of the 63 pathogens detected, 38 (60.3%) were found in both species (Figure 5), indicating the transmission of infection among wild birds. It is particularly important to note that the pathogens detected in this study were searched at the species level, but the percentage of species sequences identified by high-throughput sequencing is very small. In other words, the abundance and species of pathogenic bacteria were greatly reduced. Migratory birds are potential pathogen hosts and play an important role in the circulation and transmission of pathogenic microorganisms. Wild birds themselves carry pathogenic microorganisms that can also infect sympatric domestic poultry [43], in particular, the foraging ground of poultry and wild birds overlaps to a certain extent at Shengjin Lake. Moreover, all known flu strains that infect humans and other mammals probably originate from wild bird strains [49]. Because of human activities, such as feeding poultry in the field, the pathogens carried by birds are likely to spread to human [50]. To protect wild birds and prevent the spread of wildlife borne pathogens to human society, we must avoid the mixed group of poultry and wild birds and strengthen the research about intestinal pathogens of wild birds.

## 5. Conclusions

A significant difference was observed in the intestinal bacteria of hooded cranes and bean geese, and direct or indirect contact with each other caused the bacteria to spread among species in the mixed-species flock. Interestingly, bean geese had a greater influence on hooded cranes. In addition, we found a very high proportion of common pathogens among these two hosts. This work could help improve our understanding of the factors that influence gut bacteria in wild waterbirds and has great significance in the protection of wild birds, disease prevention, and disease control. Our study also has certain limitations. First, we only used 109 samples in our experiment. Second, our study was carried out at one wintering site rather than in multiple wintering sites simultaneously. Finally, we only used data for one year instead of multiple years. These limitations should be clarified in future studies.

## Figures and Tables

**Table 1:** Dominant bacterial phyla information in the hooded crane and bean goose.

Bacterial Phyla	Average Relative Abundance ^1^ (%)		Significance ^2^
	HC	BG	
Firmicutes	64.93 (22.30) ^a^	68.67 (24.90) ^a^	0.410
Proteobacteria	20.80 (16.02) ^a^	20.81 (18.27) ^a^	0.999
Actinobacteria	8.28 (6.76) ^a^	5.86 (6.15) ^b^	0.049
Bacteroidetes	1.77 (4.86) ^a^	1.81 (3.54) ^a^	0.723

^1^ Average relative dominant bacterial phyla abundance in the gut microbiota of the hooded cranes or bean geese; ^2^ Significance was calculated by the one-way ANOVA or Mann-Whitney U test. HC: Hooded Crane, BG: Bean Goose. Different superscript letters indicate a significant difference at *p* < 0.05.

**Table 2:** Differences in microbial composition based on ANOSIM analysis.

	Host Variable			Period Variable	
	R	*p*		R	*p*
HCE VS. BGE	0.374	0.001	HCE VS. HCM	0.748	0.001
HCM VS. BGM	0.720	0.001	HCM VS. HCL	0.429	0.001
HCL VS. BGL	0.250	0.001	BGE VS. BGM	0.327	0.001
HC VS. BG	0.259	0.001	BGM VS. BGL	0.052	0.194

HC: hooded crane; BG: bean goose; E: early period; M: middle period; L: late period.

**Table 3:** SIMPER analysis showing the contribution of bacterial taxa to the differences among groups. Taxonomic abbreviations: f, family; g, genus; s, species.

	Contribution (%)						
Taxa	HCE vs. BGE	HCM vs. BGM	HCL vs. BGL	HCE vs. HCM	HCM vs. HCL	BGE vs. BGM	BGM vs. BGL
*g__Lactobacillus*	12.83	37.02	14.61	18.29	26.28	20.68	19.86
*g__Solibacillus*	7.27	—	2.88	5.08	2.59	5.96	—
f__Peptostreptococcaceae	7.19	1.89	—	5.89	—	4.81	2.09
*g__Exiguobacterium*	3.09	—	—	—	—	—	—
*g__Streptococcus*	2.53	—	—	—	—	3.42	1.48
f__Enterococcaceae	—	4.43	—	—	—	4.88	5.22
*g__Bacillus*	—	1.49	1.03	—	2.29	—	—
*g__Agrobacterium*	—	1.39	—	—	1.56	—	—
*g__Clostridium*	—	—	4.28	—	—	—	4.60
*s__F.* *prausnitzii*	—	—	1.03	—	—	—	—
f__Nitrosomonadaceae	—	—	—	2.17	—	—	—
*g__Paenibacillus*	—	—	—	1.91	1.18	—	—

## Data Availability

The raw data have been submitted to the NCBI Sequence Read Archive (accession number SRP292607).

## Supplementary Materials

The following are available online at https://www.mdpi.com/2076-2615/11/1/233/s1, Figure S1: Study area and sample collection sites, Figure S2: Intestinal bacterial OTUs in the hooded crane and bean goose on overall, Figure S3: Intestinal bacterial alpha diversity in the hooded crane and bean goose on overall, Figure S4: LEfSe analysis of gut bacterial biomarkers in the hooded crane and bean goose, Figure S5: Intestinal bacterial OTUs in the hooded crane and bean goose in different periods, Table S1: Sampling information of the hooded crane and bean goose, Table S2: PCR system of 16 S rRNA gene in the V4-V5 region of bacteria, Table S3: Distribution of alpha-diversity and dominant phyla data of intestinal bacteria, Table S4: Intestinal bacterial and potentially pathogenic information across the samples, Table S5: Alpha-diversity differences between hooded cranes and bean geese in different periods, Table S6: Indicator OTU of the treatments (OTUs with a relative abundance of less than 0.5% are not listed), Table S7: Gut bacteria of the early period in different hosts and different periods, Table S8: Potential pathogens carried by the gut of hooded crane and bean goose.
