# Supplementary material for: Is Intestinal Bacterial Diversity Enhanced by Trans-Species Spread in the Mixed-Species Flock of Hooded Crane (Grus monacha) and Bean Goose (Anser fabalis) Wintering in the Lower and Middle Yangtze River Floodplain?
Source: Animals (Basel). 2021 Jan 19;11(1):233. doi: 10.3390/ani11010233 (PMC7832407; doi:10.3390/ani11010233)
Supplement: Supplementary file 1 [file animals-11-00233-s001.pdf]

animals

MDPI

Supplementary materials

Is Intestinal Bacterial Diversity Enhanced by Trans-Species Spread in the Mixed-Species Flock of Hooded Crane (*Grus monacha*) and Bean Goose (*Anser fabalis*) Wintering in the Lower and Middle Yangtze River Floodplain?

Zhuqing Yang [1,2] and Lizhi Zhou [1,2,*]

[1] School of Resources and Environmental Engineering, Anhui University, Hefei 230601, China; x18201023@stu.ahu.edu.cn
[2] Anhui Province Key Laboratory of Wetland Ecological Protection and Restoration (Anhui University), Hefei 230601, China
[*] Correspondence: zhoulz@ahu.edu.cn

Supplementary

Citation: Yang, Z.; Zhou, L. Is Intestinal Bacterial Diversity Enhanced by Trans-Species Spread in the Mixed-Species Flock of Hooded Crane (*Grus monacha*) and Bean Goose (*Anser fabalis*) Wintering in the Lower and Middle Yangtze River Floodplain?. *Animals* **2021**, *11*, x. https://doi.org/10.3390/xxxxx

Received: 04 January 2021
Accepted: 12 January 2021
Published: 18 January 2021

Publisher's Note: MDPI stays neutral with regard to jurisdictional claims in published maps and institutional affiliations.

Copyright: © 2021 by the authors. Submitted for possible open access publication under the terms and conditions of the Creative Commons Attribution (CC BY) license (http://creativecommons.org/licenses/by/4.0/).

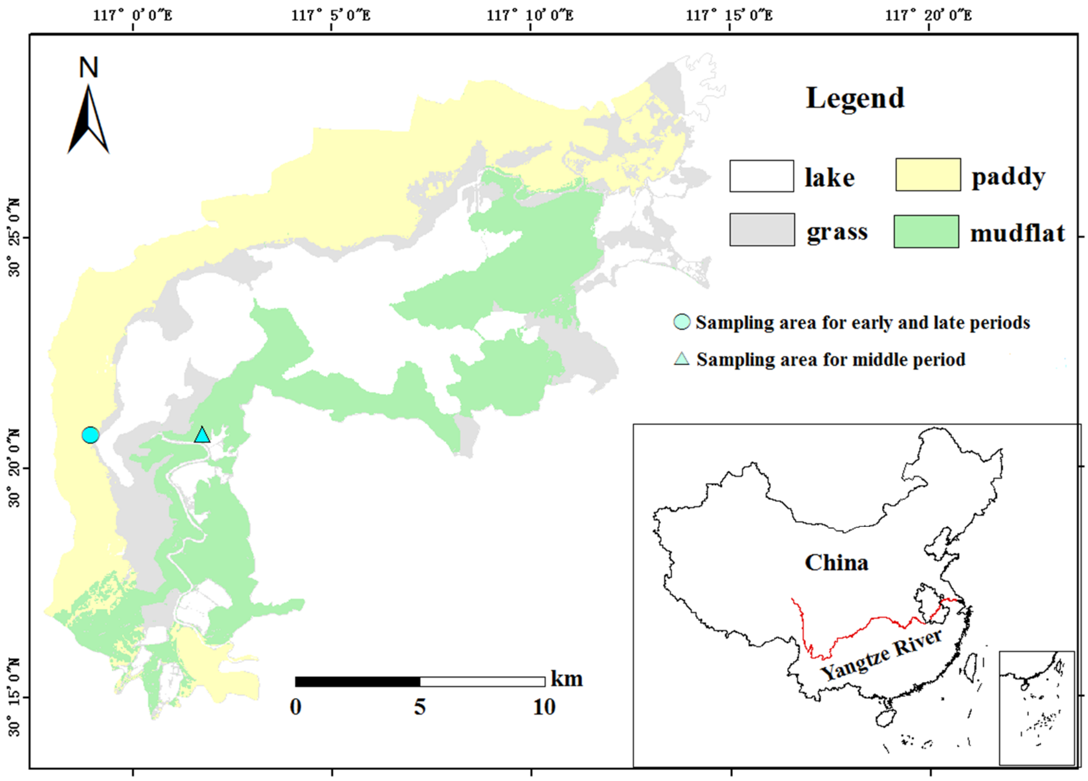

Figure S1. Study area and sample collection sites.

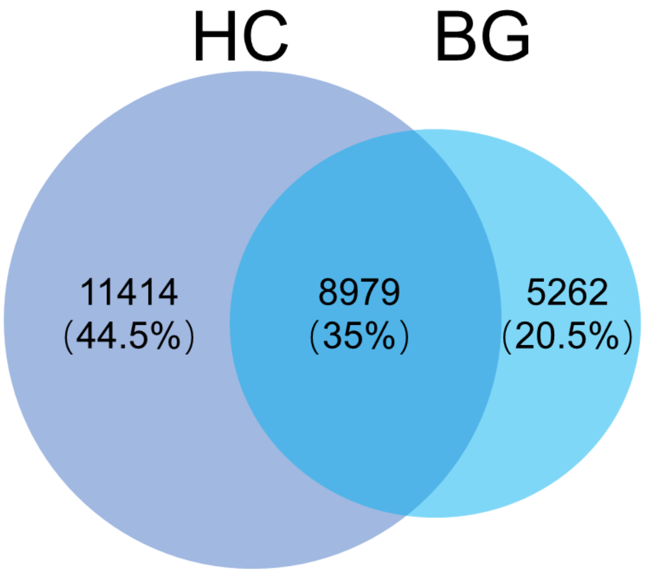

Figure S2. Intestinal bacterial OTUs in the hooded crane and bean goose on overall.HC: hooded crane; BG: bean goose.

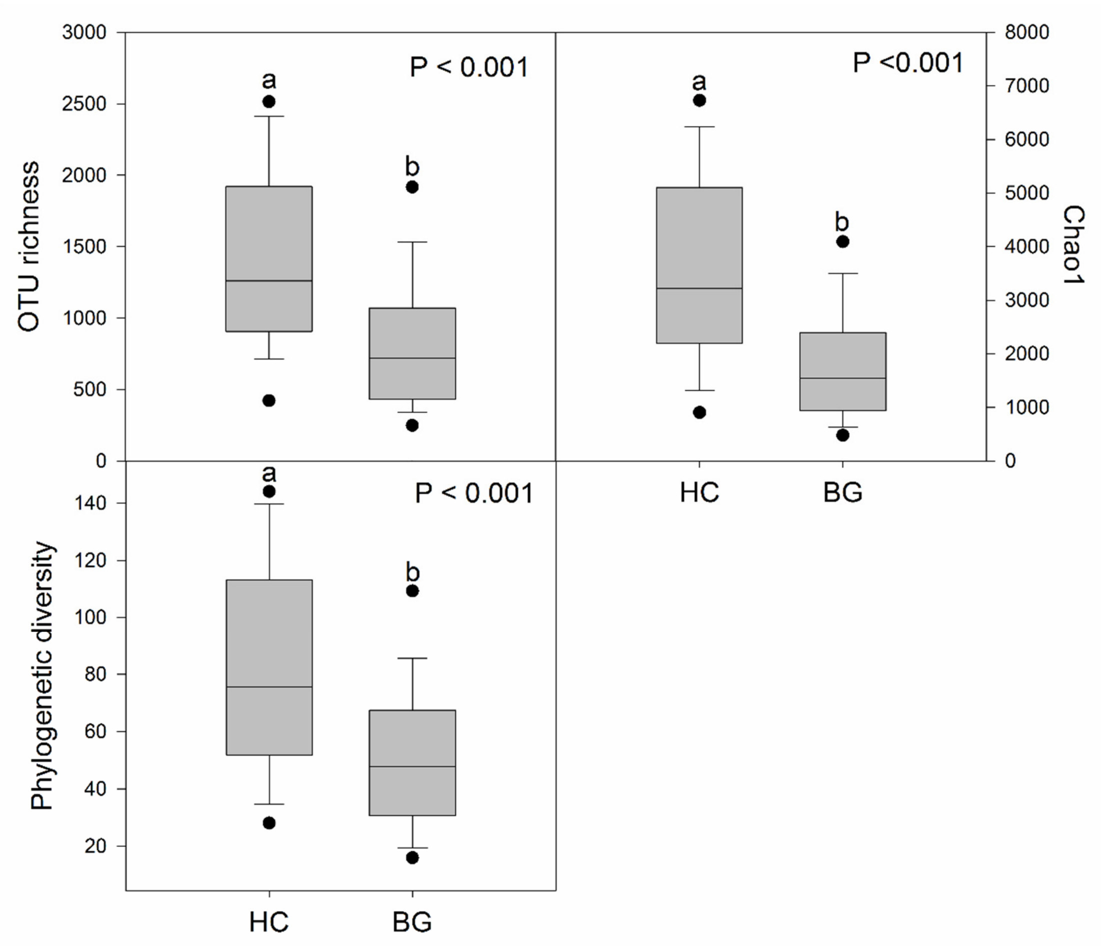

Figure S3. Intestinal bacterial alpha diversity in the hooded crane and bean goose on overall.Bars represent mean; error bars denote standard deviation; Differences of intestinal bacterial alpha diversity between different species were identified by one-way ANOVA ($P < 0.001$). HC: hooded crane; BG: bean goose.

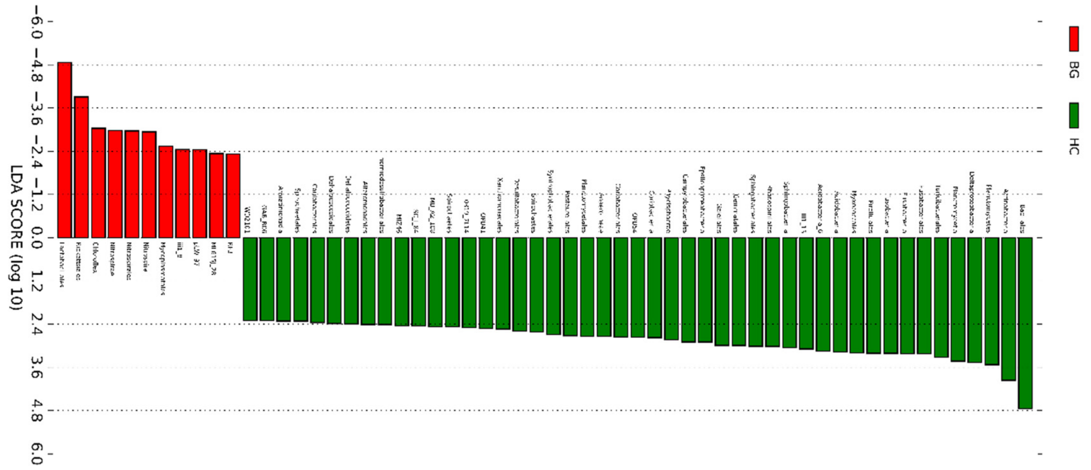

Figure S4. LEfSe analysis of gut bacterial biomarkers in the hooded crane and bean goose.Identified phylotype biomarkers ranked by effect size and the alpha value was < 0.05. red, phylotypes overrepresented in gut of bean goose; green, phylotypes overrepresented in gut of hooded crane. HC: hooded crane; BG: bean goose.

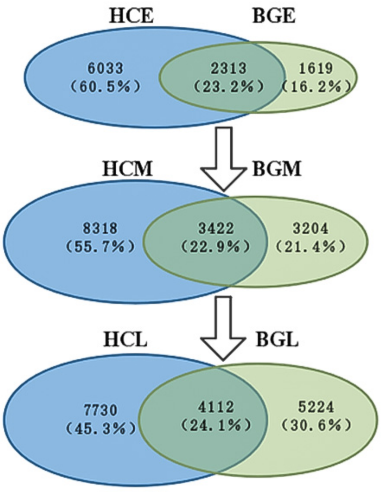

Figure S5. Intestinal bacterial OTUs in the hooded crane and bean goose in different periods.HC: hooded crane; BG: bean goose; E: early period; M: middle period; L: late period.

Table S1. Sampling information of the hooded crane and bean goose.

Wintering period	Species	Sampling time	Sampling site	Habitat	Type and number of species
Early period	Hooded crane	23rd Nov., 2018	Yangetou	Grass land	More than 100 hooded cranes
Early period	Bean goose	23rd Nov., 2018	Yangetou	Grass land	More than 300 bean goose

Middle period	Hooded crane	9th Jan., 2019	Tongxinwei	Rice field	More than 100 hooded cranes
Middle period	Bean goose	10th Jan., 2019	Tongxinwei	Grass land	200-300 bean goose
Later period	Hooded crane	8th Mar., 2019	Yangetou	Grass land	More than 100 hooded cranes
Later period	Bean goose	8th Mar., 2019	Yangetou	Grass land	200-300 bean goose

Table S2. PCR system of 16 S rRNA gene in the V4-V5 region of bacteria.

Component	Volume
5×FastPfu Buffer	4 μl
2.5 mM dNTPs	2 μl
Forward Primer[1](5 μM)	0.8 μl
Reverse Primer[2](5 μM)	0.8 μl
FastPfu Polymerase	0.4 μl
BSA	0.2 μl
Template DNA	10 ng
ddH2O	up to 20 μl

[1] Forward Primer: 515F（5′-GTGCCAGCMGCCGCGG-3′）[2] Reverse Primer: 907R（5′-GGACTACHVGGGTWTCTAAT-3′）.

Table S3. Distribution of alpha-diversity and dominant phyla data of intestinal bacteria.

**α-index and Phylum**	**Kolmogorov-Smirnov test (P value)**					
	**HCE VS. BGE**	**HCM VS. BGM**	**HCL VS. BGL**	**HCE VS. HCM .VS HCL**	BGE VS. BGM VS. BGL	HC VS. BG
Bacterial OTU richness	0.634	0.346	0.637	0.351	0.396	—
Bacterial Chao1	0.475	0.636	0.370	0.439	0.324	—
Bacterial PD	0.492	0.320	0.860	0.595	0.770	—
Pathogenic OTU richness	—	—	—	—	—	0.267
Firmicutes	—	—	—	—	—	0.071
Proteobacteria	—	—	—	—	—	0.112
Actinobacteria	—	—	—	—	—	0.027
Bacteroidetes	—	—	—	—	—	0.000

Table S4. Intestinal bacterial and potentially pathogenic information across the samples.

**Sample**	**OTUs**		Sequences		Pathogen sequences proportion(%)[1]
	**Bacteria**	Pathogen	Bacteria	Pathogen	
BGE1	414	7	8026	33	0.413
BGE3	269	1	11882	4	0.050
BGE4	1680	14	25874	31	0.388
BGE5	581	8	14735	27	0.338
BGE6	247	2	37095	2	0.025
BGE7	375	2	21539	28	0.350
BGE8	610	10	27372	14	0.175
BGE9	242	1	42009	1	0.013
BGE12	610	7	50254	55	0.688
BGE14	324	1	37631	1	0.013

	**OTUs**		Sequences		
**Sample**	**Bacteria**	Pathogen	Bacteria	Pathogen	Pathogen sequences proportion(%)[1]
BGE15	357	4	33127	13	0.163
BGE16	366	3	57082	5	0.063
BGE17	348	3	13415	64	0.800
BGE19	235	3	44413	7	0.088
BGE20	583	7	25068	35	0.438
BGM22	1020	22	35851	131	1.638
BGM23	714	10	14143	22	0.275
BGM24	648	9	35197	14	0.175
BGM25	911	15	15530	22	0.275
BGM26	790	19	58201	335	4.188
BGM27	710	17	23805	159	1.988
BGM28	410	18	46376	883	11.038
BGM29	601	26	30557	859	10.738
BGM30	596	10	21679	21	0.263
BGM31	1190	15	44640	37	0.463
BGM32	1007	17	33220	270	3.375
BGM33	1573	36	20655	496	6.200
BGM34	1086	12	19656	22	0.275
BGM35	1147	5	15008	6	0.075
BGM36	919	24	23188	110	1.375
BGM37	1212	13	56091	28	0.350
BGM38	927	11	20531	21	0.263
BGM39	510	3	17173	3	0.038
BGM40	517	5	26214	9	0.113
BGL41	2384	17	32224	28	0.350
BGL42	1477	19	22251	36	0.450
BGL43	1382	9	21224	26	0.325
BGL44	481	7	30583	189	2.363
BGL45	1151	9	32152	11	0.138
BGL46	2324	4	35123	8	0.100
BGL47	937	4	24022	10	0.125
BGL48	1095	14	39293	21	0.263
BGL49	488	5	58336	16	0.200
BGL50	1572	20	31937	35	0.438
BGL51	706	11	21438	28	0.350
BGL52	767	9	10311	13	0.163
BGL54	418	8	21049	15	0.188
BGL55	502	2	30492	2	0.025
BGL56	429	7	46017	21	0.263
BGL57	982	12	22889	448	5.600
BGL59	756	2	9984	2	0.025
BGL60	949	7	32830	39	0.488
HCE61	795	10	23751	40	0.500
HCE62	1089	19	19226	71	0.888
HCE63	1053	12	13157	59	0.738
HCE64	852	10	35748	85	1.063
HCE65	1200	18	31760	40	0.500
HCE66	1213	14	36331	51	0.638
HCE67	1513	24	31389	70	0.875

	**OTUs**		Sequences		
**Sample**	**Bacteria**	Pathogen	Bacteria	Pathogen	Pathogen sequences proportion(%)[1]
HCE68	651	5	15352	125	1.563
HCE69	315	11	8197	107	1.338
HCE70	1999	25	14874	108	1.350
HCE71	1267	20	9795	102	1.275
HCE72	1075	14	24608	61	0.763
HCE73	951	19	35991	100	1.250
HCE74	821	8	29649	32	0.400
HCE75	2537	42	21741	269	3.363
HCE76	861	12	30953	33	0.413
HCE77	1333	22	26908	118	1.475
HCE78	754	11	31864	93	1.163
HCE79	1310	17	21234	54	0.675
HCE80	842	12	26264	50	0.625
HCM81	1407	15	29302	72	0.900
HCM82	1505	11	35677	30	0.375
HCM83	906	13	45516	55	0.688
HCM84	1244	10	42846	22	0.275
HCM85	1183	12	16228	34	0.425
HCM86	1666	20	20986	33	0.413
HCM87	1103	4	38426	12	0.150
HCM88	1007	10	38185	20	0.250
HCM89	903	20	38114	52	0.650
HCM90	2682	18	28969	36	0.450
HCM91	1754	26	37085	39	0.488
HCM92	1868	19	34900	40	0.500
HCM93	2221	13	38239	51	0.638
HCM94	2501	16	33322	93	1.163
HCM95	1095	11	16661	26	0.325
HCM96	1426	10	14689	47	0.588
HCM97	1256	19	38848	62	0.775
HCM98	924	13	32923	46	0.575
HCM99	295	2	31972	3	0.038
HCM100	780	9	49423	13	0.163
HCL101	2441	20	15106	83	1.038
HCL102	971	29	23337	127	1.588
HCL105	435	4	18072	6	0.075
HCL107	531	15	9609	147	1.838
HCL108	2028	10	34374	93	1.163
HCL109	2361	37	9035	185	2.313
HCL110	1834	13	32055	22	0.275
HCL111	2505	20	27702	132	1.650
HCL112	2207	18	32465	36	0.450
HCL113	2485	10	17720	47	0.588
HCL114	2068	17	16353	33	0.413
HCL115	1920	12	15643	25	0.313
HCL116	2292	15	34186	93	1.163
HCL117	1839	9	8258	12	0.150
HCL118	1625	6	38028	13	0.163
HCL119	1659	6	22936	12	0.150

	**OTUs**		Sequences		
**Sample**	**Bacteria**	Pathogen	Bacteria	Pathogen	Pathogen sequences proportion(%)[1]
HCL120	1563	23	25729	95	1.188

[1] Pathogenic sequences proportion(%): The pathogenic sequence of the sample/minimum sequence read depth.

Table S5. Alpha-diversity differences between hooded cranes and bean geese in different periods.

	**One-Way ANOVA（P value）**		
	**HCE VS. BGE**	HCM VS. BGM	HCL VS. BGL
OUT richness	< 0.001	0.004	< 0.001
Phylogenetic diversity	0.003	0.003	< 0.001
Chao1	< 0.001	< 0.001	< 0.001

Table S6. Indicator OTU of the treatments (OTUs with a relative abundance of less than 0.5% are not listed).

**Treatment**	**Indicator OTU**	**Taxonomy**	**P value**	**Relative abundance%**	**Subordinate to the species/period**
Early	39419	g__Lactobacillus	0.002	16.581	BG
	18419	g__Lactobacillus	0.048	6.344	HC
	35844	g__Paenibacillus	0.001	1.211	HC
	21576	g__Streptococcus	0.003	1.048	BG
	26998	g__Agrobacterium	0.001	0.863	HC
	4877	f__Clostridiaceae	0.021	0.759	HC
	28476	s__M. adhaesivum	0.001	0.658	HC
	1435	o__SJA-22	0.001	0.506	HC
Middle	39419	g__Lactobacillus	0.001	16.581	BG
	18419	g__Lactobacillus	0.001	6.344	HC
	38898	g__Solibacillus	0.001	4.400	HC
	19095	f__Enterococcaceae	0.001	1.537	BG
	34322	g__Bacillus	0.001	1.293	HC
	26575	g__Clostridium	0.011	1.214	HC
	35844	g__Paenibacillus	0.001	1.211	HC
	21576	g__Streptococcus	0.002	1.048	BG
	32658	g__Solibacillus	0.001	1.025	HC
	4877	f__Clostridiaceae	0.047	0.759	HC
	15421	g__Paenibacillus	0.001	0.602	HC
Late	18419	g__Lactobacillus	0.003	6.344	HC
	26309	f__Peptostreptococcaceae	0.034	3.745	HC
	34322	g__Bacillus	0.001	1.293	HC
	35844	g__Paenibacillus	0.001	1.211	HC
	21576	g__Streptococcus	0.004	1.048	HC
	32658	g__Solibacillus	0.008	1.025	HC
	26998	g__Agrobacterium	0.002	0.863	BG
	661	f__Enterobacteriaceae	0.01	0.797	BG
	38024	f__Clostridiaceae	0.001	0.617	HC
	15421	g__Paenibacillus	0.002	0.602	HC
	1435	o__SJA-22	0.001	0.506	BG
HC	39419	g__Lactobacillus	0.001	16.581	Late
	18419	g__Lactobacillus	0.001	6.344	Middle
	38898	g__Solibacillus	0.003	4.400	Early
	26309	f__Peptostreptococcaceae	0.001	3.745	Early

Treatment	Indicator OTU	Taxonomy	P value	Relative abundance%	Subordinate to the species/period
	19095	f__Enterococcaceae	0.001	1.537	Late
	26575	g__Clostridium	0.014	1.214	Late
	35844	g__Paenibacillus	0.016	1.211	Early
	21576	g__Streptococcus	0.006	1.048	Late
	32658	g__Solibacillus	0.002	1.025	Early
	26998	g__Agrobacterium	0.003	0.863	Middle
	19076	f__Clostridiaceae	0.001	0.790	Early
	4877	f__Clostridiaceae	0.001	0.759	Early
	40120	g__Exiguobacterium	0.001	0.732	Early
	23342	f__Clostridiaceae	0.001	0.716	Early
	28476	s__M. adhaesivum	0.001	0.658	Early
	1435	o__SJA-22	0.001	0.506	Early
BG	39419	g__Lactobacillus	0.031	16.581	Middle
	38898	g__Solibacillus	0.008	4.400	Early
	19095	o__Lactobacillales	0.001	1.537	Middle
	34322	g__Bacillus	0.022	1.293	Early
	26575	g__Clostridium	0.001	1.214	Late
	35844	g__Paenibacillus	0.01	1.211	Early
	32658	g__Solibacillus	0.007	1.025	Early
	26998	g__Agrobacterium	0.013	0.863	Late
	19076	f__Clostridiaceae	0.003	0.790	Early
	40120	g__Exiguobacterium	0.001	0.732	Early
	15421	g__Paenibacillus	0.001	0.602	Late
	1435	o__SJA-22	0.003	0.506	Late

Taxonomic leaves: p, phylum; c, class; o, order; f, family; g, genus; s, species. Acronym: HC, hooded crane; BG, bean goose.

Table S7. Gut bacteria of the early period in different hosts and different periods.

	Average sequences					
	BGE	BGM	BGL	HCE	HCM	HCL
BGE-specific bacteria	302.47	310.05	225.39	0.00	126.55	264.94
HCE- specific bacteria	0.00	584.11	936.72	1033.75	1114.80	824.06
BGE bacteria	8000.00	6810.37	5987.28	6966.25	6052.90	6226.65
HCE bacteria	7697.53	7084.42	6698.61	8000.00	7041.15	6785.76

Table S8. Potential pathogens carried by the gut of hooded crane and bean goose.

Pathogentic species	Symptom	Infect target	Content in HC	Content in BG
Agrobacterium vitis	Crown Galls	Grape	1539	237
Clostridium perfringens	Tissue necrosis, Bacteremia, etc	human, birds, etc	634	97
Haemophilus parainfluenzae	Haemophilus parainfluenzae, endocarditis	human, birds, etc	280	17
Enterococcus cecorum	Vertebral osteomyelitis, etc	Human, poultry, birds, etc	233	2983
Prevotella copri	Rheumatoid arthritis, Colitis	Human, mouse	147	995
Rhodococcus fascians	Destruction of gene, leafy gall syndrome	Plant	141	12
Escherichi coli	Enterocolitis, bacteremia	Humans, birds, livestock	80	3

Pathogentic species	Symptom	Infect target	Content in HC	Content in BG
Flavobacterium succinicans	bacterial gill disease	fish	63	5
Pantoea agglomerans	septicemia	human	55	43
Streptococcus minor	peritonitis	human	48	16
Mucispirillum schaedleri	Inflammation, etc	mouse	47	14
Rhodococcus ruber	Keratitis	human	45	14
Capnocytophaga ochracea	Sepsis, Endocarditis, etc	human	35	36
Helicobacter pylori	Stomach ache, Nausea, etc	human	28	12
Ruminococcus gnavus	gall bladder perforation, diverticulitis	human	21	16
Vermamoeba vermiformis	Corneal damage, etc	Humans, fish	20	28
Enterococcus casseliflavus	Endometritis, etc	Humans, horses	19	43
Actinomadura vinacea	chronic, multifocal, pyogranulomatous dermatitis	mammals	19	7
Rhodococcus globerulus	Bacteremia, hepatitis	human	16	2
Sphingobacterium multivorum	Bacteremia,Acute Meningitis	human	16	1
Bacillus Clausii	septicemia	human	13	2
Elizabethkingia meningoseptica	Meningitis, bacteriuria	human	13	—
Macrococcus caseolyticus	Inflammatory infiltration, etc	Chicken, mouse	12	1
Propionibacterium acnes	acne inflammation	human	11	9
Treponema amylovorum	rheumatoid arthritis, osteoarthritis	human	7	—
Flavobacterium columnare	Columnaris, etc	fish	7	1
Bacillus horikoshii	tetrodotoxin-producing	human, animal	5	2
Clostridium intestinale	Microorganism	human	5	49
Aureimonas altamirensis	Tissutis, peritonitis, myeloma, empyema	human	4	3
Paracoccus aminovorans	promoted the in vitro growth of V. cholerae	human	4	1
Avibacterium gallinarum	valvular endocarditis	human	4	—
Brevibacillus laterosporus	Inhibit egg hatching, etc	invertebrate	4	—
Clostridium neonatale	necrotizing enterocolitis	human	4	—
Peptostreptococcus anaerobius	Bacteremia	horses	3	3
Bacillus anthracis	Meningitis	human	3	—
Brevundimonas diminuta	Pleuritis	human	3	—
Porphyromonas endodontalis	Periapical periodontitis, etc	human	3	2
Lactococcus garvieae	Septicemia, etc	human, fish, cattle, etc	2	12
Mycoplasma hyorhinis	Polyserositis	pig	2	—
Acinetobacter johnsonii	cellulitis	fish	2	2
Bacillus megaterium	Brain Abscess	human	2	1

Pathogentic species	Symptom	Infect target	Content in HC	Content in BG
Methylobacterium mesophilicum	Meningitis	human	2	—
Piscirickettsia salmonis	Piscirickettsiosis, etc	fish	2	—
Rothia aeria	Sepsis、Endocarditis	human	1	—
Mycobacterium arupense	Tenosynovitis, osteomyelitis	human	1	—
Pseudoclavibacter bifida	bacteremia, chronic obstructive pulmonary	human	1	—
Mycobacterium celatum	Pneumonia, lymphadenitis, skin infection	Human, mammal	1	—
Aliivibrio fischeri	brill Scophthalmus rhombus	fish	1	1
Streptomyces lanatus	Streptomyces	human	1	1
Eggerthella lenta	Bacteremia	human	1	1
Acinetobacter lwoffii	Red Head Disease,gastroenteritis	human, fish	1	—
Flexispira rappini	bacteremia、variable immunodeficiency	human	1	1
Plesiomonas shigelloides	Diarrhea	human	1	—
Pseudomonas stutzeri	Necrotizing Pneumonia, aortic valve endocarditis	human	1	—
Pseudomonas viridiflava	Bacterial Leaf Spot	Plant	1	31
Agromyces mediolanus	Peritonitis, bacteremia	human	—	4
Prevotella nigrescens	Periodontal Disease,etc	human	—	2
Bacteroides uniformis	Spontaneous spondylodiscitis	human	—	1
Treponema socranskii	Endodontic infections, etc	human	—	1
Legionella jamestowniensis	fatal pneumonia	human	—	1
Grimontia hollisae	Gastroenteritis, Septicaemia	human	—	1
Bacillus cereus	Nausea,Vomiting, etc	human	—	1
Bacteroides caccae	Bloodstream Infection	human	—	1
